# Diagnostic Accuracy of Non-Invasive Imaging for Detection of Colonic Inflammation in Patients with Inflammatory Bowel Disease: A Systematic Review and Meta-Analysis

**DOI:** 10.3390/diagnostics11101926

**Published:** 2021-10-18

**Authors:** Meshari T. Alshammari, Rebecca Stevenson, Buraq Abdul-Aema, Guangyong Zou, Vipul Jairath, Shellie Radford, Luca Marciani, Gordon W. Moran

**Affiliations:** 1 Department of Diagnostic Radiology, College of Applied Medical Sciences, University of Hail, Hail 55473, Saudi Arabia; 2 Translational Medical Sciences and National Institute for Health Research (NIHR) Nottingham Biomedical Research Centre, Nottingham University Hospitals NHS Trust and University of Nottingham, Nottingham NG7 2UH, UK; shellie.radford1@nottingham.ac.uk (S.R.); luca.marciani@nottingham.ac.uk (L.M.); gordon.moran@nottingham.ac.uk (G.W.M.); 3 Precision Imaging Beacon, University of Nottingham, Nottingham NG7 2UH, UK; rebecca.stevenson@nottingham.ac.uk; 4 East Midlands North Deanery, Queen’s Medical Centre, Nottingham University Hospitals NHS Trust, Nottingham NG7 2UH, UK; buraqheider@yahoo.com; 5 Department of Epidemiology & Biostatistics, Schulich School of Medicine & Dentistry, University of Western Ontario, London, ON N6A 5C1, Canada; gy.zou@alimentiv.com (G.Z.); vipul.jairath@alimentiv.com (V.J.); 6 Department of Medicine, Schulich School of Medicine & Dentistry, University of Western Ontario, London, ON N6A 5C1, Canada

**Keywords:** inflammatory bowel disease, Crohn’s disease, ulcerative colitis, colon, endoscopy, ultrasonography, magnetic resonance imaging

## Abstract

Endoscopy is the gold standard for objective assessment of colonic disease activity in inflammatory bowel disease (IBD). Non-invasive colonic imaging using bowel ultrasound (US), computed tomography (CT), and magnetic resonance imaging (MRI) may have a role in quantifying colonic disease activity. We reviewed the diagnostic accuracy of these modalities for assessment of endoscopically or histopathologically defined colonic disease activity in IBD. We searched Embase, MEDLINE, and the Web of Science from inception to 20 September 2021. QUADAS-2 was used to evaluate the studies’ quality. A meta-analysis was performed using a bivariate model approach separately for MRI and US studies only, and summary receiver operating characteristic (ROC) curves were obtained. CT studies were excluded due to the absence of diagnostic test data. Thirty-seven studies were included. The mean sensitivity and specificity for MRI studies was 0.75 and 0.91, respectively, while for US studies it was 0.82 and 0.90, respectively. The area under the ROC curves (AUC) was 0.88 (95% CI, 0.82 to 0.93) for MRI, and 0.90 (95% CI, 0.75 to 1.00) for US. Both MRI and US show high diagnostic accuracy in the assessment of colonic disease activity in IBD patients.

## 1. Introduction

The global incidence of Inflammatory Bowel Disease (IBD) is rising, raising the disease prevalence to 0.3% [1]. A considerable amount of IBD patients have colonic involvement, so objective assessment of colonic inflammation is paramount for diagnosis, monitoring, and clinical management [2].

A treat-to-target approach is advocated to ensure best long-term outcomes in IBD patients [3]. Current recommendations based on the Selecting Therapeutic Targets in IBD (STRIDE) program recommend an objective assessment as a target rather than symptom resolution alone [4]. This is defined as an absence of ulceration for Crohn’s disease (CD) and an endoscopic Mayo score of 0 or 1 for ulcerative colitis (UC) on ileo-colonoscopy. However, ileo-colonoscopy has limitations. It requires bowel preparation, can be uncomfortable, is invasive and associated with rare but potentially serious risks such as perforation [5]. Only 60% of patients rate ileo-colonoscopy as an acceptable experience, with only 75% willing to undergo the procedure repeatedly [6]. Moreover, after the procedure, patients require a recovery period especially if needing sedation.

Non-invasive imaging tools using magnetic resonance imaging (MRI), computed tomography (CT), or ultrasonography (US) are widely available, well tolerated [7], require less intensive bowel preparation, and are routinely used to assess small bowel inflammation [8,9]. However, their utility in assessing colonic inflammation is less clear. Diagnostic accuracy data relating to non-invasive colonic imaging are very limited. We systematically searched the literature for studies reporting the diagnostic accuracy of non-invasive colonic imaging, where either ileo-colonoscopy or histopathology was used as the gold-standard technique and summarized our findings using meta-analyses.

## 2. Materials and Methods

### 2.1. Search Methods, Types of Studies, and Participants

A systematic literature review was undertaken to investigate the diagnostic accuracy of non-invasive colonic imaging in both CD and UC. The preferred reporting items for systematic reviews and meta-analyses (PRISMA) [10] guidelines were followed. The search strategy was based on the patients, intervention, comparator, and outcomes (PICO) framework model [11] (Table 1), and is summarized in Table S1 (Supplementary Materials) together with the search terms. The search was conducted through three different databases: Embase, MEDLINE, and the Web of Science, which were searched from inception to 20 September 2021. To avoid missing relevant references that might be excluded from the search results in some databases, additional related studies from citation chaining of reviews and meta-analysis were identified, and manually included.

Pediatric and adult IBD patients were included, without age limit. We included randomized controlled trials, and retrospective and prospective cross-sectional studies including both case-control type accuracy studies and cohort type accuracy studies. Exclusion criteria were animal or in vitro studies, studies not reported in English language, case reports, reviews or systemic literature reviews, editorials and opinion pieces, meta-analysis, and conference abstracts.

### 2.2. Index Tests and Target Conditions

Studies that examined the accuracy of non-invasive colonic imaging in IBD for detecting endoscopic or histologically active UC or CD as a target condition were eligible. No restriction was placed on the type of scoring systems that were used for the reference standard.

### 2.3. Data Collection and Analysis

Study selection was performed in two phases, after removing duplicate results in EndNote X9 software (Clarivate Analytics, Philadelphia, PA, USA). The first phase involved screening and filtering titles and abstracts of search results against inclusion and exclusion criteria by two reviewers (MA and LM). During the second phase, two reviewers (LM and GM) independently assessed eligibility of full-text manuscripts of the studies identified, recording the reasons for exclusions. Any discrepancies between the reviewers were resolved through discussion, until consensus was reached. A PRISMA flowchart [10] summarizing the outcomes of this process was created. The review protocol was registered with the International Prospective Register of Systematic Reviews (PROSPERO, CRD42020183914).

### 2.4. Risk of Bias Assessment

The quality of the studies was evaluated by two reviewers (MA, BA) independently using a quality assessment tool for diagnostic accuracy studies (QUADAS-2) [12]. The QUADAS tool involves 4 key domains that consider patient selection, index test, reference standard, and flow of patients through the study and timing of the index tests and reference standard (flow and timing).

### 2.5. Statistical Analysis and Data Synthesis

Raw data were extracted from the included studies in the form of a 2 × 2 table, including the total number of segments as well as the numbers of true positives (TP), false positives (FP), true negatives (TN), and false negatives (FN). The Revman software version 5.4 (Review Manager, the Cochrane Collaboration, Oxford, UK) was used to calculate TP, FP, TN, and FN from reported sensitivity and specificity values if these were not immediately available in the published literature.

A meta-analysis of diagnostic accuracy of both MRI and US raw data was conducted using R “mada” package version 0.5.10 (R: A language and environment for statistical computing. R Foundation for Statistical Computing, Vienna, Austria). Receiver operating characteristic (ROC) curves were produced to depict the relationship between individual and summarized values of specificity and sensitivity. Study heterogeneity was assessed using the I^2^ statistic. Sensitivity analyses using the random-effects model for between-subgroup comparisons were conducted for the same raw data, excluding studies using histopathology rather than endoscopic disease activity as the reference standard and by IBD type, excluding UC studies due to insufficient number.

## 3. Results

### 3.1. Results of the Search

**Figure 1:**
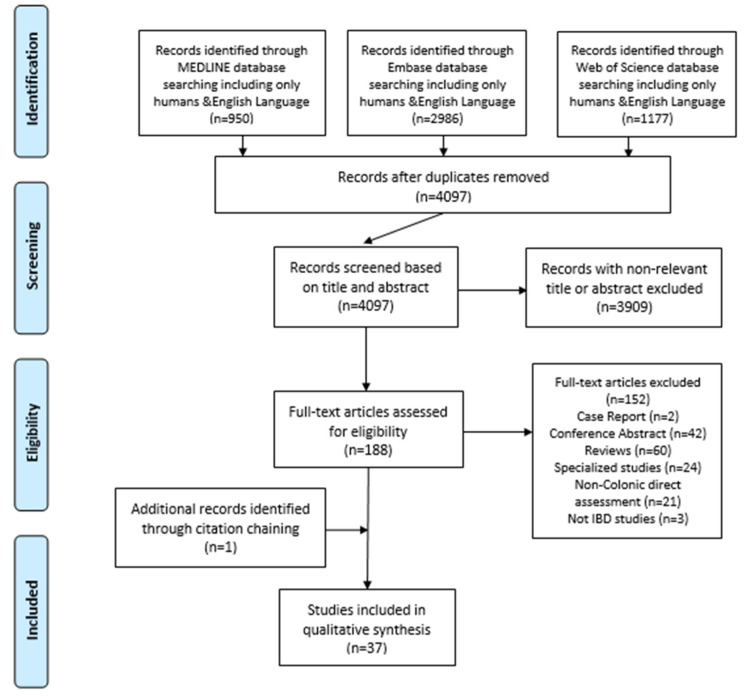
Flow diagram of the systematic review search. Adapted from Moher et al. (2009) and preferred reporting items for systematic reviews and meta-analyses (PRISMA) [10].

The literature search on 20 September 2021 yielded a total of 5113 publications from three databases. After removing duplicate records, 4097 publications remained, which were screened by title and abstract. This led to the exclusion of 3909 publications and the inclusion of 188 publications for full-text assessment. One hundred and fifty-two publications were excluded thereafter. Only one additional publication was included using citation chaining. In total, 37 studies were included in the systematic review, as shown in the PRISMA flow diagram (Figure 1) and summarized in Table S2 (Supplementary Materials).

These studies involved 23 prospective and 14 retrospective studies, investigating CD in 20 studies, UC in 4 studies, and both CD and UC in 13 studies. Multiple disease scoring systems were used. The modified Baron score was used in three studies [13,14,15], the Simple Clinical Colitis Activity Index (SCCAI) in two studies [13,16], the CD Endoscopic Index of Severity (CDEIS) in two studies [17,18], Crohn’s Disease Activity Index (CDAI) in three studies [19,20,21], Pediatric Crohn’s Disease Activity Index (PCDAI) in one study [22], Mayo Endoscopic Subscore (MES) in two studies [16,23], Truelove and Witts score in two studies [21,24], and the Simple Endoscopic Score for CD (SES-CD) was used in four studies [13,25,26,27] The performance of MRI and US was assessed in 24 and 17 studies, respectively, while CT was assessed only in two studies hence excluding this imaging modality from the meta-analyses. The diagnostic test values (TP, FP, TN, and FN) of the colonic segments and the sensitivity and specificity values were not presented in all the included studies. The meta-analyses were carried out only in two separate groups, including 13 MRI studies [13,14,17,25,28,29,30,31,32] and 5 US studies [14,25,32,33], which they had either the calculated TP, FP, TN, and FN or the reported sensitivity and specificity values presented.

### 3.2. Risk of Bias Assessment

**Figure 2:**
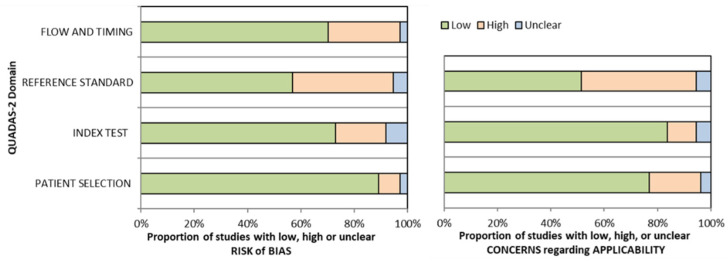
QUADAS-2 assessment of bias and applicability (graphical summary).

The results of the QUADAS-2 bias and applicability assessment are summarized in Figure 2, while Table S3 (Supplementary Materials) documents the individual bias scores for the seven domains for all included studies. The QUADAS-2 assessment showed low/intermediate/risk of bias in a large proportion of the studies across the domains. Bias in patient selection was low for 33 studies and high in 3 studies, while the flow and timing was high risk in 10 studies and low in the rest of the included studies. Due to absence of blinding in the methodology, the risk of bias in the index test and reference standard was high in 6 studies and 15 studies, respectively. Moreover, the absence of any attempt at central reading in endoscopic, radiological, and histopathological scoring introduces variability and bias within the data set, which might lead to a heterogeneity across all modalities. In some of these studies, the risks of bias in patient selection, the index test, reference standard, and flow and timing were mostly high [22,34,35]. The index test results were not interpreted without knowledge of the results of the reference standard, and the lack of blinding or unclear blinding of endoscopists was observed in many different studies [14,16,29,32,34,35,36,37,38,39,40,41]. In the majority of the studies, the timing between the index test and the reference standard varied significantly [34,35,38,41], making the diagnostic accuracy findings less homogenous [42]. Three studies did not have a consecutive or random sample of patients enrolled [34,38,40].

### 3.3. Diagnostic Accuracy for MRI

**Figure 3:**
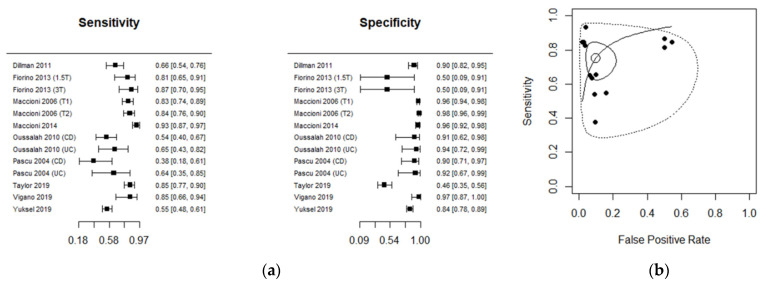
(**a**) Forest plot and (**b**) the summary receiver operating characteristic (ROC) curve illustrating the summary operating point for the diagnostic performance of MRI.

The diagnostic performance of MRI studies is presented in Table 2. Ileo-colonoscopy was used as a reference standard in 11 studies and histopathology in 2 studies. Diagnostic accuracy was investigated in CD in 11 studies and in UC in 2 studies. The performance estimates of MRI are depicted in a Forest plot (Figure 3a), with related summary receiver operating characteristic (ROC) curves (Figure 3b). The estimated mean sensitivity of the 13 combined studies was 0.75 (0.65; 0.83), whereas the specificity was 0.91 (0.83; 0.95). The area under the ROC curve (AUC) was 0.88 (95% CI, 0.82 to 0.93). The diagnostic odds ratio of each MRI study is shown in Figure S1a (Supplementary Materials).

### 3.4. Diagnostic Accuracy for US

**Figure 4:**
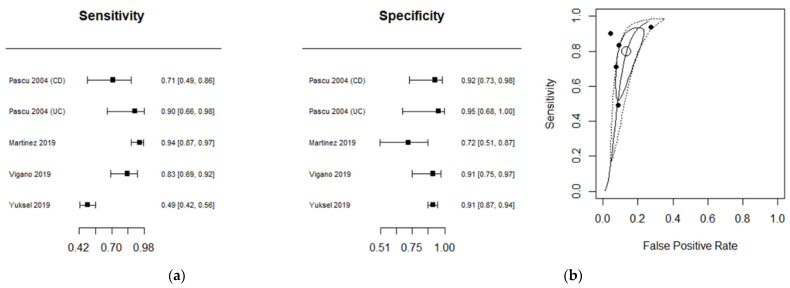
(**a**) Forest plot and (**b**) the summary receiver operating characteristic (ROC) curve illustrating the summary operating point for the diagnostic performance of US.

The diagnostic performance of US studies is presented in (Table 3). Ileo-colonoscopy was used as a reference standard in four studies and histopathology in a single study. Diagnostic accuracy was investigated in CD in four studies and in UC in a single study. The performance estimates of US are depicted in a Forest plot (Figure 4a), with related summary ROC curves (Figure 4b). The estimated mean sensitivity based on the five combined studies was 0.82 (0.62; 0.92), whereas the specificity was 0.90 (0.87; 0.93). The area under the ROC curve was 0.9 (95% CI, 0.75 to 1.00). The diagnostic odds ratio of each US study is shown in Figure S1b (Supplementary Materials).

### 3.5. Between-Study Heterogeneity

The pooled analysis revealed a significant variation between MRI studies, which was attributable to heterogeneity rather than chance (Sensitivity *I*^2^ = 86.5%, Specificity *I*^2^ = 88.4%, *p* < 0.0001). For US studies, the sensitivity analysis revealed a significant variation between studies (*I*^2^ = 86.7%, *p* < 0.0001). At the same time, there was no indication of heterogeneity in the specificity based on tau^2^ (*I*^2^ = 0.0%, *p* = 0.1810).

### 3.6. Sensitivity Analyses

Subgroup analysis was performed for CD studies only. The pooled estimates of mean sensitivity and specificity including nine MRI studies were 0.77 (0.64; 0.86) and 0.89 (0.75; 0.95), respectively, while the estimated mean sensitivity and specificity based on four US studies were 0.79 (0.56; 0.92) and 0.90 (0.86; 0.93), respectively.

**Figure 5:**
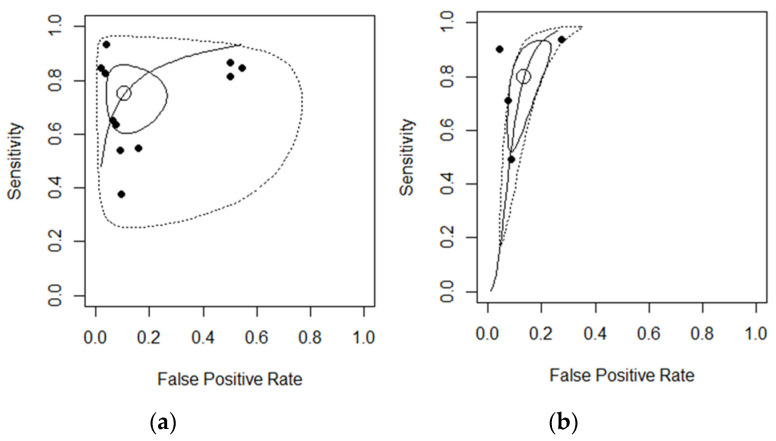
(**a**) The summary receiver operating characteristic (ROC) curve of MRI studies excluding two histopathology studies, (**b**) and summary ROC curve of US studies excluding one histopathology study.

To further explore the reasons of heterogeneity, another subgroup analysis was performed excluding studies using histopathology as the reference standard. The performance estimates of studies based solely on ileo-colonoscopy are represented in the summary ROC curve (MRI in Figure 5a and US in Figure 5b). The pooled estimates of mean sensitivity and specificity based on the 11 MRI studies were 0.75 (0.64; 0.84) and 0.90 (0.79; 0.95), respectively, while the area under the ROC curve was 0.86 (95% CI, 0.80, 0.92) after excluding two histopathology studies. The estimated mean sensitivity and specificity based on the four US studies was 0.82 (0.56; 0.94) and 0.90 (0.87; 0.93), respectively, while the AUC after excluding one histopathology study was 0.89 (95% CI, 0.74 to 1.00).

## 4. Discussion

Symptomatic and endoscopic remission is the recommended treatment target proposed by regulatory bodies [43,44], and in every day clinical IBD practice [4]. Ileo-colonoscopy is invasive, uncomfortable, costly, and associated with complications. To minimize patient impact, other biomarkers, such as C-reactive protein and fecal calprotectin have been developed and play a role in disease monitoring, but due to their relative lack of sensitivity and correlation to disease extent and location [45], endoscopy remains the gold standard for disease monitoring. Moreover, no other modality can provide histological sampling or facilitate colorectal cancer surveillance.

In this study, we incorporated data from different studies and found that the pooled sensitivity estimate was fair (76% for MRI and 82% for US). Concomitantly, the specificity of both diagnostic modalities was excellent (91%), indicating a robust capacity for both MRI and US to discriminate disease-free patients from those with active disease. This was further corroborated by the pooled accuracy estimates of 88% for MRI and 90% for US. The proximity of the combined estimate to the upper left corner of both ROCs (Figure 3 and Figure 5) emphasizes the ability of MRI and US to discriminate between endoscopic healing and colonic inflammation. After excluding the histopathology studies, the pooled accuracy estimates in the subgroup analyses of MRI and US were 86% and 89%, respectively. These findings may potentially highlight a role for US- and MRI-based non-invasive colonic imaging in predicting endoscopic-free remission in both CD and UC.

US has a major advantage of being able to provide a point-of-care assessment of colonic disease activity that would facilitate and expedite decision making and may improve disease outcomes. The accuracy, sensitivity, and specificity of US for CD recurrence were estimated to be 91%, 94%, and 72%, respectively [33]. In newly diagnosed patients with CD, US had higher sensitivity of 67% in regard to colonic CD presence when compared to MRI (47%) [31]. US identified abnormal bowel segments in 41 out of 115 patients, which were not visible on ileo-colonoscopy [46]. In addition, the sensitivity of US in the detection of stricturing disease in patients with CD was 88%. These findings probably highlight the ability of US to detect transmural inflammation, an inherent limitation of endoscopy.

MRI can be a useful tool in assessing mucosal healing and treatment response in patients with UC using 1.5T MRI platforms [16]. Combining standard MRI data sequences with diffusion weighted imaging (DWI) may be useful in assessing colonic inflammation in patients with UC, even without using oral contrast or rectal preparation. DWI has shown the same accuracy as a post-contrast sequence for the evaluation of endoscopic inflammation, which may substitute using gadolinium injection in detecting colonic inflammation [13]. MRI with a water-based enema can be used to assess disease activity in the colon in patients who are not suitable for colonoscopy. This technique was utilized and tolerated by the entire patient cohort who were investigated by Boraschi et al. [35].

MRI, including T2 weighted imaging (T2W), has shown to be accurate in the evaluation of colonic CD lesions [29]. The comparison between T2W and T1W post-contrast sequences showed the same high accuracy, ranging between 93% to 95% in detecting colonic CD [30], with these findings being replicated in another cohort [47]. These results provide further support to the possibility of performing contrast-free MRE, hence removing gadolinium-related risks, such as allergy, renal dysfunction, and potential long-term deposits in the central nervous system. Moreover, T2W images to date have all been mainly 2D and qualitatively assessed. Quantitative T2W image is a step-change that now allows more objective and reproducible disease assessments that should enhance the performance of MRI in the non-invasive measurement of colonic inflammation [48,49].

There are several limitations to this study that should be acknowledged. In this study, studies with different methodological design were pooled: retrospective, prospective, cohort studies, and cross-sectional studies. The meta-analysis did not include PET/MRI studies, which assessed the inflammation in IBD. However, many of the studies analyzed were not designed to look only at colonic imaging. The majority of studies specifically pertaining to US are retrospective in design, with only one study having a large prospective multi-center design setting [9]. Most studies were undertaken in CD with only a handful in UC, and none compared the diagnostic accuracy across the various colonic segments. Due to the relatively small UC sample size, both disease types were grouped in a single IBD cohort. However, a subgroup analysis was carried out for the CD cohort only, including MRI and US studies separately, which showed almost the same specificity for MRI and US compared to the single IBD cohort analysis, while no significant differences were found in the sensitivity (77% for MRI and 79% for US). Moreover, endoscopic, US, and MRI assessments are heterogenous, with a lack of central reading and using a number of partially validated scores that may limit the reproducibility of the findings across all modalities. Similarly, histopathological assessment was undertaken by local pathologists and without using any validated scoring systems [28,32,34,50]. Histopathology scoring using validated measures was not available for all studies. Nevertheless, a subsequent sensitivity analysis precluding histopathology scoring did not have a major effect on the diagnostic accuracy of both US and MRE. There were risks of bias from the studies included. The major risks were related to a lack of blinding or unclear blinding of endoscopists, lack of a consecutive or random sample of patients enrolled, and a lack of an appropriate interval between imaging and endoscopy. Moreover, only two studies investigated the use of CT for colonic disease assessment, hence limiting the meta-analysis to MRI and US studies.

Histological remission in UC patients has been considered a predictor of sustainable corticosteroid-free remission, and has been associated with reduced hospitalization and surgeries [51]. Some of the studies analyzed used this as a gold standard of disease activity [16], but this was in a minority. Future studies aiming to validate the role of non-invasive imaging in the assessment of colonic inflammation need to have a prospective design, initially with a single center setting and consequently with a multi-center design, with further work streams assessing inter-observer variability, repeatability, reproducibility, and reversibility of such measures [52]. Quantitative T2W imaging, together with 3D or multiple slice imaging and more automated analyses will invariably decrease bias and variability within these readouts. Furthermore, such studies should aim to investigate the performance of such platforms in CD and UC separately, while undertaking further analyses to investigate the effect of segmental disease location in the colon on overall diagnostic accuracy.

## 5. Conclusions

In conclusion, both MRI and US have shown good diagnostic accuracy in the assessment of colonic inflammation in IBD patients. These non-invasive imaging tools could be used to monitor disease activity and response to therapy in IBD patients, especially in cases where colonoscopy is incomplete or not possible to be performed.

## Figures and Tables

**Table 1:** Patients, intervention, comparator, and outcomes (PICO).

PICO Framework	
Participants	Human (without any age limit)
Interventions	Non-invasive colonic imaging, such as MRI, CT, and US
Comparator	Colonoscopy or histology
Outcomes	Measuring colonic inflammation

**Table 2:** Raw data of the included MRI studies, including true positives (TP), false positives (FP), false negatives (FN), true negatives (TN), sensitivity (95% confidence intervals, CI), specificity (95% confidence intervals, CI), MRI platform used, disease cohort studies (CD or UC), and reference standard used.

Author	No. of Segments	TP	FP	FN	TN	Sensitivity (95% CI)	Specificity (95% CI)	MRI	Disease	Ref Standard
Dillman 2011 [28]	149	44	8	23	74	0.65 [0.53; 0.76]	0.90 [0.81; 0.95]	1.5T	CD	Histopathology
Fiorino 2013 (1.5T) [17]	34	26	1	6	1	0.81 [0.63; 0.92]	0.50 [0.01; 0.98]	1.5T	CD	Ileo-colonoscopy
Fiorino 2013 (3T) [17]	32	26	1	4	1	0.86 [0.69; 0.96]	0.50 [0.01; 0.98]	3T	CD	Ileo-colonoscopy
Maccioni 2006 (T1) [30]	413	85	11	18	299	0.82 [0.73; 0.89]	0.96 [0.93; 0.98]	1.5 T1	CD	Ileo-colonoscopy
Maccioni 2006 (T2) [30]	413	87	6	16	304	0.84 [0.76; 0.90]	0.98 [0.95; 0.99]	1.5 T2	CD	Ileo-colonoscopy
Maccioni 2014 [29]	300	112	7	8	173	0.93 [0.87; 0.97]	0.96 [0.92; 0.98]	1.5T	CD	Ileo-colonoscopy
Oussalah 2010 (CD) [13]	61	27	1	23	10	0.54 [0.39; 0.68]	0.90 [0.58; 0.99]	1.5T	CD	Ileo-colonoscopy
Oussalah 2010 (UC) [13]	36	13	1	7	15	0.65 [0.40; 0.84]	0.93 [0.69; 0.99]	1.5T	UC	Ileo-colonoscopy
Pascu 2004 (CD) [14]	37	6	2	10	19	0.37 [0.15; 0.64]	0.90 [0.69; 0.98]	1.5T	CD	Ileo-colonoscopy
Pascu 2004 (UC) [14]	24	7	1	4	12	0.63 [0.30; 0.89]	0.92 [0.63; 0.99]	1.5T	UC	Ileo-colonoscopy
Taylor 2019 [31]	186	89	44	16	37	0.84 [0.76; 0.91]	0.45 [0.34; 0.57]	1.5T	CD	Ileo-colonoscopy
Vigano 2019 [32]	65	22	1	4	38	0.84 [0.65; 0.95]	0.97 [0.86; 0.99]	1.5T	CD	Histopathology
Yuksel 2019 [25]	426	123	32	101	170	0.54 [0.48; 0.61]	0.84 [0.78; 0.88]	1.5T	CD	Ileo-colonoscopy

TP = true positive; FP = false positive; FN = false negative; TN = true negative.

**Table 3:** Raw data of the included US studies including true positives (TP), false positives (FP), false negatives (FN), true negatives (TN), sensitivity (95% confidence intervals, CI), specificity (95% confidence intervals, CI), disease cohort studies (CD or UC), and reference standard used.

Author	No. of Segments	TP	FP	FN	TN	Sensitivity (95% CI)	Specificity (95% CI)	IBD Type	Ref Standard
Pascu 2004 (CD) [14]	37	13	1	5	18	0.7222 [0.4652; 0.9031]	0.9474 [0.7397; 0.9987]	CD	Ileo-colonoscopy
Pascu 2004 (UC) [14]	24	13	0	1	10	0.9286 [0.6613; 0.9982]	1.0000 [0.6915; 1.0000]	UC	Ileo-colonoscopy
Jesus Martinez 2019 [33]	108	84	5	5	14	0.9438 [0.8737; 0.9815]	0.7368 [0.4880; 0.9085]	CD	Ileo-colonoscopy
Vigano 2019 [32]	65	32	2	6	25	0.8421 [0.6875; 0.9398]	0.9259 [0.7571; 0.9909]	CD	Histopathology
Yuksel 2019 [25]	426	94	20	97	215	0.4921 [0.4192; 0.5653]	0.9149 [0.8716; 0.9472]	CD	Ileo-colonoscopy

TP = true positive; FP = false positive; FN = false negative; TN = true negative.

## Data Availability

All data in this study were obtained from already published material in scientific journals, referenced in the paper, and can be obtained by any individual with access to these.

## Supplementary Materials

The following are available online at https://www.mdpi.com/article/10.3390/diagnostics11101926/s1, Figure S1: Diagnostic odds ratio (DOR) of MRI and US across all studies, Table S1: Search strategy that used in the systematic review, Table S2: Search strategy that used in the systematic review, Table S3: QUADAS-2 summary of risk and applicability (tabular format). References [53,54,55,56,57] are cited in the Supplementary Materials.
