# Supplementary material for: Diagnostic Accuracy of Non-Invasive Imaging for Detection of Colonic Inflammation in Patients with Inflammatory Bowel Disease: A Systematic Review and Meta-Analysis
Source: Diagnostics (Basel). 2021 Oct 18;11(10):1926. doi: 10.3390/diagnostics11101926 (PMC8534724; doi:10.3390/diagnostics11101926)
Supplement: Supplementary file 1 [file diagnostics-11-01926-s001.zip › diagnostics-1430563-supplementary.pdf]

diagnostics

MDPI

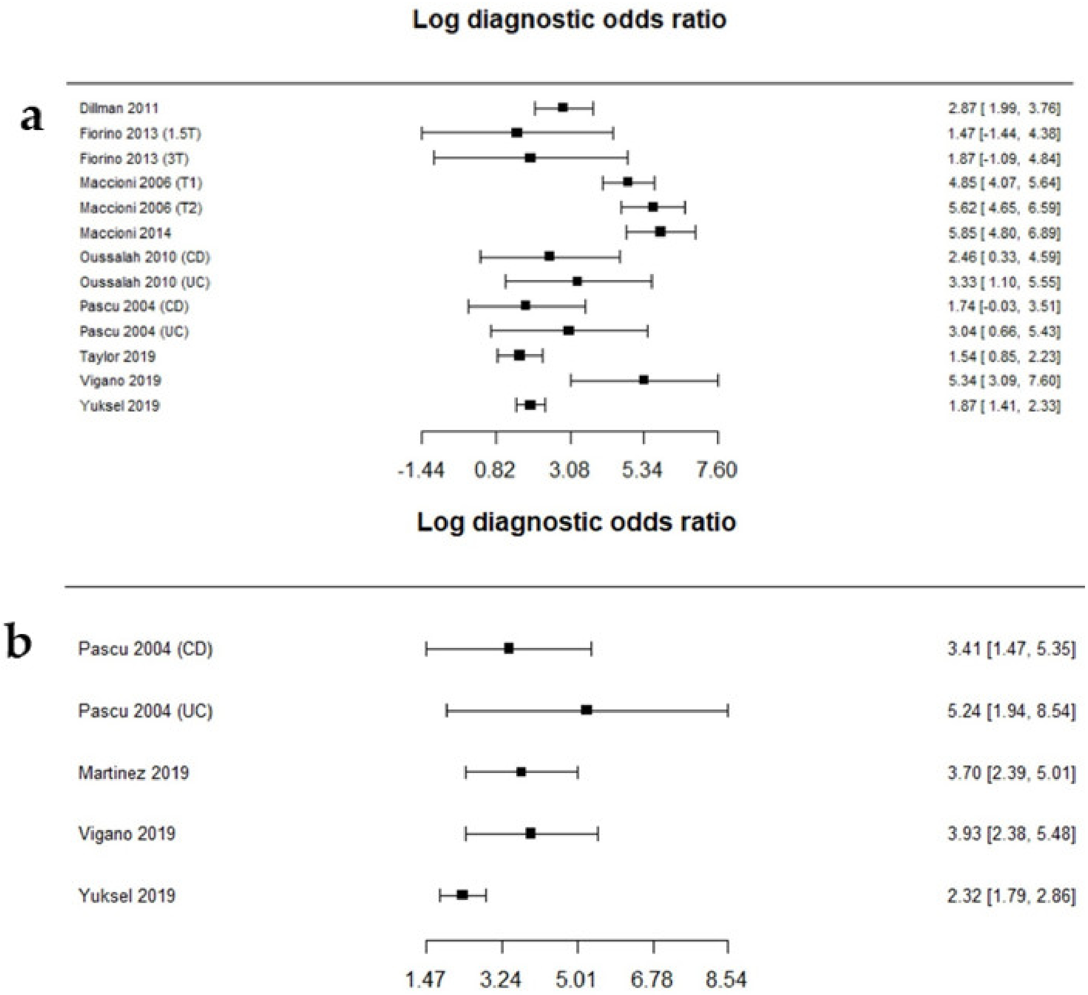

Figure S1. (**a**) Diagnostic odds ratio (DOR) of MRI across all studies, (**b**) Diagnostic odds ratio (DOR) of US across all studies.

Table S1. Search strategy that used in the systematic review.

Order	Ovid Platform (EMBASE & MEDLINE)	Web of Science
1	("Inflammatory Bowel Disease*" OR ibd).ti,ab.	TS = ("Inflammatory Bowel Disease*" OR ibd)
2	exp Inflammatory Bowel Diseases/	TS = (colon* NEAR/3 inflam*)
3	(colon* ADJ3 inflam*).ti,ab.	TS = (colon* NEAR/3 imag*)
4	(colon* ADJ3 imag*).ti,ab	TS = (Colitis or UC)
5	exp Colitis/	TS = (colon or rectum or ileum or "large intestine")
6	(colitis or UC).ti,ab	TS = (crohn? or CD)
7	(colon or rectum or ileum or "large intestine").ti,ab	TS = ("magnetic resonance" or "magnetic resonance enterography" or "MR colonography" or MRI or MR or "MR imag*")
8	(crohn? or CD).ti,ab.	TS = ("computed tomograp*" or ct)
9	("magnetic resonance" or "magnetic resonance enterography" or "MR colonography" or MRI or "MR imag*").ti,ab	TS = (ultraso* or ultrasonography)

**10**	magnetic resonance imaging/ or diffusion magnetic resonance imaging/ or magnetic resonance imaging, cine/ or multiparametric magnetic resonance imaging/	#1 or #2 or #3 or #4 or #5 or #6
**11**	exp Tomography, X-Ray Computed/	#7 or #8 or #9
**12**	("computed tomograp*" or ct).ti,ab	#10 and #11
**13**	exp Ultrasonography/	TS = ("Sensitivity and Specificity")
**14**	(ultraso* or ultrasonography).ti,ab.	TS = ((pre-test or pretest) NEAR probability)
**15**	1 or 2 or 3 or 4	TS = (post-test probability)
**16**	5 or 6 or 7 or 8	TS = (predictive value$)
**17**	9 or 10	TS = (likelihood ratio$)
**18**	11 or 12	#13 or #14 or #15 or #16 or #17
**19**	13 or 14	#12 and #18
**20**	15 or 16	TI = (animal or "in vitro" or review or "meta analysis" or rat or mouse)
**21**	17 and 20	#19 NOT #20 and English
**22**	18 and 20	
**23**	19 and 20	
**24**	21 and 22 and 23	
**25**	limit 24 to (English language and humans)	

Table S2. Summary table of the diagnostic studies summarized all the available quantification data of the diagnostic studies, started with retrospective studies and followed by prospective studies – smallest to the biggest.

Study ID	IBD Type (n)	Age	Study Design	Reference Standard	Aims and Outcomes	Disease Scoring System	Oral Contrast	Modality/Manufacturers	Accuracy (%)	Sensitivity (%)	Specificity (%)	Modality Protocol
**Dagia 2008**	CD (28)	-	Retrospective	Endoscopy and examination under anaesthesia and where available surgery, barium studies, CT and ultrasound	To review 3-T MRI in the evaluation of Crohn disease in a paediatric population and to assess the correlation of different imaging methods	-	Sorbitol (1500-2000 mL)	3T MRI (Siemens TIM TRIO, Erlangen, Germany)	-	-	-	mainly involved T2W and T1W pre-contrast and post-contrast sequences
**Laurent 2016**	UC (29)	Median age 43	Retrospective	Endoscopy (Mayo endoscopic subscore)	to investigate [for the first time] the accuracy of the Nancy score in diagnosing MH in patients with UC. The secondary objective was to assess the Nancy score's responsiveness to change in patients	Simple Clinical Colitis Activity Index (SCCAI)	-	1.5T MRI (Signa HDx scanner (GE Healthcare, Waukesha, WI, USA)	73.0	75.0	67.0	Involved DWI

Study ID	IBD Type (n)	Age	Study Design	Reference Standard	Aims and Outcomes	Disease Scoring System	Oral Contrast	Modality/Manufacturers	Accuracy (%)	Sensitivity (%)	Specificity (%)	Modality Protocol
					treated for active UC							
**Dillman 2011**	CD (31)	6-17	Retrospective	Histopathology (within two months of having the MRE)	Detecting CD lesions	-	VoLumen (E-Z-EM/Bracco Imaging, Princeton, N.J.)	MRI 1.5T (Achieva scanner (Philips Healthcare, Best, The Netherlands),	-	66.0	90.0	T2W, axial DWI, fat-saturated T2W and fat-saturated T1W
**Campari 2017**	(31) CD&UC	(range: 5-17 years)	Retrospective	Ileocolonoscopy and histology	to determine the diagnostic performance of standard MR enterography in detecting colonic inflammation.	-	20 ml/kg of 0.5% solution of Macrogol 4000 + (a dose of 0.1 mmol/kg of gadoterate meglumine (Dotarem; Guerbet, Villepinte, France)	MRI 1.5-T scanner (Achieva; Philips)	88.0	94.0	64.0	BTFE axial<br>BTFE coronal<br>T2 SS TSE axial<br>T2 SS TSE coronal<br>T2 SS TSE SPAIR axial<br>T2 SS TSE SPAIR coronal<br>DWIBS[b] (b=1,000) axial<br>DWIb (b=0, 50, 300, 800) axial<br>THRIVE axial<br>THRIVE coronal
**Kamel 2020**	Total 40<br>UC (14)<br>CD (26)	>18	Retrospective	colonoscopy	to compare the role of MRE and bowel ultrasound in diagnosis and follow-up of inflammatory bowel disease	UC: Truelove and Witts classification. CD: Crohn's Disease Activity	Ingestion of 1 to 2 L of hyperosmolar oral contrast was performed for about 45 min before	US<br>&<br>1.5T MRI with IV contrast (Achieva, Philips Medical System, Best, Netherlands	US: 70.0	US: 37.5	US: 91.7	MRI protocol: Coronal T2 SSFSE, Coronal SSFP, Axial T2WI, Axial DWI, 3D-THRIVE, Axial post contrast fat-suppressed gradient-echo T1WI, Coronal post contrast fat-

Study ID	IBD Type (n)	Age	Study Design	Reference Standard	Aims and Outcomes	Disease Scoring System	Oral Contrast	Modality/Manufacturers	Accuracy (%)	Sensitivity (%)	Specificity (%)	Modality Protocol
					(IBD) patients in Egypt.	Index (CDAI).	the magnetic resonance (MR) exam started.					suppressed gradient-echo T1WI
**Cansu 2020**	CD (45)	18 to 72 years	Retrospective	Colonoscopy and histopathology	to investigate the potential use of T2-weighted sequences with diffusion weighted imaging (DWI) in magnetic resonance (MR) enterography instead of conventional contrast-enhanced MR imaging (MRI) sequences for the evaluation of active inflammation in Crohn disease.	Crohn disease endoscopic index of severity (CDEIS)	The patients were given 1500mL 3% mannitol solution (500mL every 15 minutes) for intestinal distention 45 minutes before the scan. Further, 20mg hyoscine N-butylbromide (Buscopan, Boehringer Ingelheim, czacibasi, Turkey) was	3T MRI Magnetom, Skyra; Siemens Healthcare, Erlangen, Germany	95.5	88.7	97.9	axial T2-weighted half- Fourier single-shot fast spin-echo sequence, coronal T2-weighted HASTE, axial fat suppressed T2-weighted HASTE, coronal T2-weighted steady-state gradient echo sequence, axial DWI (with b factors of 50, 400, and 800 s/mm2, T1-weighted 3-dimensional (3D) gradient echo sequences with fat suppression in the arterial, portal venous, and late venous phase on the coronal plane and in the late venous phase on the axial plane T1-weighted 3D gradient echo.

Study ID	IBD Type (n)	Age	Study Design	Reference Standard	Aims and Outcomes	Disease Scoring System	Oral Contrast	Modality/Manufacturers	Accuracy (%)	Sensitivity (%)	Specificity (%)	Modality Protocol
							administered intravenously before MR enterography to decrease intestinal peristalsis.					
**Barber 2017**	CD or indeterminate colitis (49)	7 to 17 years	Retrospective	Endoscopy and histopathology	To investigate concordance of bowel ultrasound and magnetic resonance enterography (MRE) in identifying active disease in children with inflammatory bowel disease	-	A 2.5% mannitol solution was orally ingested over a 1 hour period prior to the study. Intravenous gadolinium was given.	US 1.5T MRI (Ingenia - Philips, Amsterdam, Netherlands).	-	-	MRE 85.1 US 86.6	The standard sequence set includes axial and coronal balanced fast field echo (FFE) and single-shot fast spin echo. Coronal two-dimensional (2D) gradient-echo T1-weighted fat-saturated images were acquired pre-contrast medium administration, with coronal post-contrast images at 30 seconds and 70 seconds, and axial post-contrast images of the whole abdomen at 90 seconds. Diffusion-

Study ID	IBD Type (n)	Age	Study Design	Reference Standard	Aims and Outcomes	Disease Scoring System	Oral Contrast	Modality/Manufacturers	Accuracy (%)	Sensitivity (%)	Specificity (%)	Modality Protocol
												weighted imaging (DWI) was acquired up to b=600.
**Liu 2021**	CD (66)	18 to 77 years	Retrospective	endoscopy	to develop an ultrasound consolidated score (UCS) in determining the activity of Crohn's disease (CD) and evaluate it with reference to simple endoscopic score (SES).	simple endoscopic score (SES)	On the day of US examination, approximately 1,000ml of 2.5% isotonic mannitol was orally administered to each patient 60min before US examination.	US	92.3	88.3	95.5	-
**Johnson 2009**	CD and UC (70)	-	Retrospective	Colonoscopy	To determine if computed tomographic enterography is a sensitive and specific method for detecting Crohn colitis and ulcerative colitis	-	The CTE examination was performed by the administration of water-attenuation oral contrast	CT	-	74.0	95.0 in poorly distended colons	-

Study ID	IBD Type (n)	Age	Study Design	Reference Standard	Aims and Outcomes	Disease Scoring System	Oral Contrast	Modality/Manufacturers	Accuracy (%)	Sensitivity (%)	Specificity (%)	Modality Protocol
							material (VoLumen, EZEM, Lake Success, NY, USA), 450 mL administered at 60, 40, and 20 min before CT scanning.					
**Kim 2019**	Total 77<br>CD (44)<br>UC (8)<br>Indeterminate IBD (6)	7 to 18 years	Retrospective	Endoscopy and histopathology	To determine whether gadolinium-based contrast agents are necessary in assessing active bowel inflammation on MRE in pediatric patients with known or suspected IBD.	-	IV contrast (Magnevist and Dotarem)	1.5T MRI Signa HDXT 23.0 scanner (GE Healthcare, Milwaukee, WI)	-	67.0 vs. 67.0	80.0 vs. 68.0	Coronal T2 SSFSE BH<br>Axial T2 SSFSE BH<br>Axial T2 SSFSE FS BH<br>Axial 2-D FIESTA BH (non FS)<br>Coronal 2-D FIESTA BH (non FS)<br>Axial DWI b=200, b=800, b=1,000<br>Axial T1 FSPGR FS BH<br>Intravenous contrast agent administered<br>Coronal LAVA FS DYNAMIC BH ASSET

Study ID	IBD Type (n)	Age	Study Design	Reference Standard	Aims and Outcomes	Disease Scoring System	Oral Contrast	Modality/Manufacturers	Accuracy (%)	Sensitivity (%)	Specificity (%)	Modality Protocol
												Axial T1 FSPGR FS BH + contrast Coronal LAVA FS BH + contrast
**Thierry 2018**	CD (96)	adult	Retrospective	Endoscopy (CDEIS)	to assess the accuracy of the Nancy score in detecting mucosal healing and treatment response, and predicting surgery, in an independentc ohort of CD patients.	-	1500 mL polyethylene glycol-electrolyte (PEG) solution	MRI (Signa HDx scanner (GE Healthcare, Waukesha, WI, USA)	75.0	70.0	80.0	The protocol comprised a two-dimensional [2D], single-shot [SS], fast spin-echo, short echo time sequence in the axial and coronal planes, a 2D SS free precession [FP] sequence with fat saturation in the coronal plane, a diffusion-weighted sequence in the axial plane, a three-dimensional [3D] gradient echo T1 sequence after intravenous administration of 0.2 ml/kg body weight gadoteric acid [DOTAREM, 0.5 mmol/ml; Guerbet, Villepinte, France] at a rate of 3 mL/s for a dynamic
									85.0	92.0	68.0	

Study ID	IBD Type (n)	Age	Study Design	Reference Standard	Aims and Outcomes	Disease Scoring System	Oral Contrast	Modality/Manufacturers	Accuracy (%)	Sensitivity (%)	Specificity (%)	Modality Protocol
												study in the axial plane [with the arterial phase 25 s after injection, the portal phase 70 s after injection, and the post-equilibrium phase 2 min after injection], and a 2D gradient echo sequence with fat saturation at 3 min and 5 min after injection in the axial and coronal planes, respectively. The diffusion-weighted sequence employed a diffusion factor b of 600 s/mm², and provided two sets of images [one for the expected b value and another for a b value = 0]. The acquisition was performed in the axial plane using a 36-cm field of view, a 7-mm slice thickness with no

Study ID	IBD Type (n)	Age	Study Design	Reference Standard	Aims and Outcomes	Disease Scoring System	Oral Contrast	Modality/Manufacturers	Accuracy (%)	Sensitivity (%)	Specificity (%)	Modality Protocol
												gap, and a 335-mm2 in-plane resolution. Two stations were needed to cover the abdomen and the pelvis. The b value was set to 600 s/mm2 because this was the best compromise between the signal-to-noise ratio and lesion detection sensitivity on our MRI system. The dynamic 3D gradient echo T1 sequences after the intravenous injection of contrast medium, the 2D gradient echo T1 sequences, and the SS FP sequences were acquired with a breath-hold technique. All other sequences were performed with respiratory triggering.
**Wilkens 2016**	CD (115)	40.2 ± 13.8	Retrospective	Ileocolonoscopy	post-operative CD	Rutgeerts' score	--	US (Philips iU22 (Philips, Bothell,	-	-	-	-

Study ID	IBD Type (n)	Age	Study Design	Reference Standard	Aims and Outcomes	Disease Scoring System	Oral Contrast	Modality/Manufacturers	Accuracy (%)	Sensitivity (%)	Specificity (%)	Modality Protocol
		37.5 ± 13.7						WA) or a Toshiba Aplio XG (Toshiba, Tokyo, Japan).				
**Schreyer 2011**	207 patients (175 CD and 32 UC)	14 to 77	Retrospective	No reference standard was used	To compare the results of high-resolution ultrasound (HR-US) and magnetic resonance enterography (MRE) examinations in patients with inflammatory bowel disease (IBD)	-	1500-2000 mL of water mixed with methylcellulose	US (Sonoline Elegra (Siemens, Erlangen, Germany), Logiq 9 (General Electric, Solingen, Germany) or EUB-8500 (Hitachi, Tokyo, Japan), both using 3.5 MHz and 5 to 10 MHz high-resolution transducers). 1.5T MRI (Magnetom Symphony and Magnetom Sonata, Siemens Healthcare, Erlangen, Germany)	-	-	-	-
**Paparo 2012**	CD (221)	mean age of 50.2	Retrospective	Histology	to assess the prevalence of disease distribution, behavior, anastomotic	-	2000 mL of Isocolan (Bracco, Milan, Italy)	CT enterography	-	-	-	-

Study ID	IBD Type (n)	Age	Study Design	Reference Standard	Aims and Outcomes	Disease Scoring System	Oral Contrast	Modality/Manufacturers	Accuracy (%)	Sensitivity (%)	Specificity (%)	Modality Protocol
					recurrence and extraintestinal manifestations detected by an original CTE technique							
**Kawai 2019**	18 CD and 2 UC	-	Prospective	Computed tomography/magnetic resonance images	to evaluate the feasibility to use real-time virtual sonography (RVS) for the examination of the intestine.	-	-	US using real-time virtual sonography (RVS) - (HI VISION Ascendus; Hitachi Aloka Medical, Tokyo, Japan)	-	-	-	-
**Podgorska 2021**	UC (20)	20 to 47 years	Prospective	Endoscopy and histopathology	To determine the efficacy of intravoxel incoherent motion (IVIM) in assessing inflammatory activity in UC	Mayo endoscopic subscore (MES)	-	3T MRI (Magnetom Skyra, Siemens Medical Solutions, Erlangen, Germany)	67.0	82.0	59.0	The short protocol used for scanning consisted of unenhanced breath-hold half-Fourier acquisition single-shot turbo spin echo T2-weighted images (HASTE) in the axial plane and two DWI free-breathing using a single shot spin-echo echo

Study ID	IBD Type (n)	Age	Study Design	Reference Standard	Aims and Outcomes	Disease Scoring System	Oral Contrast	Modality/Manufacturers	Accuracy (%)	Sensitivity (%)	Specificity (%)	Modality Protocol
												planar imaging (SE-EPI) sequences: with b-values of 0, 10, 30, 50, 75, 100, 150, 200, 500, and 900 s/mm2 for IVIM, using the spectral attenuated inversion recovery fat suppression technique.
**Boraschi 2016**	(22) CD&UC	-	Prospective	conventional colonoscopy (when complete) with histopathological analysis, and surgery (when performed)	to assess the usefulness of MR colonography with a faecal tagging technique and water-based enema in patients with suspected or known inflammatory bowel disease.	-	To obtain sufficient bowel distension, a rectal enema tube was placed, and the colon was filled with 2–2.5 L of warm water using hydrostatic pressure on the MR examination table immediately before the	MRI a 1.5-T device (Symphony; Siemens Medical Systems, Erlangen, Germany)	-	-	-	a true fast imaging with steady-state precession (true-FISP) (Fig. 1b) sequence (TR/TE 4.5 ms/2.25 ms; slice thickness 4–5 mm; interslice gap 10–20 %; phase FoV 380–440 mm; matrix 256 × 256; one signal average; acquisition time 18–24 s) in the coronal and axial plane with and without fat saturation. As a second step, coronal and axial T2w half-Fourier single-shot

Study ID	IBD Type (n)	Age	Study Design	Reference Standard	Aims and Outcomes	Disease Scoring System	Oral Contrast	Modality/Manufacturers	Accuracy (%)	Sensitivity (%)	Specificity (%)	Modality Protocol
							MR examination					turbo spin-echo (HASTE) (Fig. 1c) images (TR/TE 1900 ms/95 ms; slice thickness 4–5 mm; interslice gap 10–20 %; phase FoV 380–440 mm; matrix 173 × 192; one signal average; acquisition time 18–24 s) were acquired. Subsequently, a coronal 3D T1w spoiled gradient-echo image was acquired before and 60 s after the intravenous administration
**Yu 2015**	UC (23)	-	Prospective	Endoscopy (modified Baron score)	to determine the optimal b value of colon DWI to detect colonic inflammation in patients with UC without bowel preparation at 3T, to evaluate	The modified Baron score	-	MRI (3.0 T Philips scanner (Achieva 3.0T, YX, Best, Holland).		66.2	89.7	(1) axial and coronal balanced turbo field echo with and without fat suppression [repetition time (TR), 3.4 ms; echo time (TE), 1.4 ms; matrix, 224 × 224; flip angle, 45°;

Study ID	IBD Type (n)	Age	Study Design	Reference Standard	Aims and Outcomes	Disease Scoring System	Oral Contrast	Modality/Manufacturers	Accuracy (%)	Sensitivity (%)	Specificity (%)	Modality Protocol
					the accuracy of DWI combined with MRI, and to investigate the changes in ADC of patients with UC							slice thickness, 6 mm; gap, 0 mm]; (2) axial and coronal T2-weighted single-shot fast spin echo with and without fat suppression (TR, 2000 ms; TE, 40 ms; matrix, 256 × 256; slice thickness, 6 mm; gap, 0 mm); (3) a 3D fast field echo (FFE) T1 sequence after intravenous administration of 0.2 mL/kg body weight of gadopentetatedimeglumine (Magnevist, Bayer, Germany) at a rate of 2 mL/s for a dynamic study of the axial plane with an arterial phase (25 s after injection) and a portal phase (70 s after injection) and

Study ID	IBD Type (n)	Age	Study Design	Reference Standard	Aims and Outcomes	Disease Scoring System	Oral Contrast	Modality/Manufacturers	Accuracy (%)	Sensitivity (%)	Specificity (%)	Modality Protocol
												a 2D FFE with fat saturation at 3 min after injection in axial and coronal planes; and (4) axial and/or coronal diffusion-weighted images (b = 0, 400, 600, 800 and 1000 s/mm2 ; TR, 2357 ms; TE, 62 ms; matrix, 300 × 231; slice thickness, 5 mm; gap, 0 mm; number of signals acquired, the field of view ranged between 32 and 40 cm. Acquisition time for the DWI sequences covering the abdomen and the pelvis ranged from 3 min to 5 min
**Ponorac 2021**	CD (24)	3.5 to 18 years	Prospective	histopathology	To evaluate the diagnostic performance of contrast-enhanced US in the	-	The second-generation US contrast agent (SonoVue; Bracco,	US	87.5	72.2	100	

Study ID	IBD Type (n)	Age	Study Design	Reference Standard	Aims and Outcomes	Disease Scoring System	Oral Contrast	Modality/Manufacturers	Accuracy (%)	Sensitivity (%)	Specificity (%)	Modality Protocol
					estimation of Crohn disease activity in children		Milan, Italy) in a dose of 0.05 mL/kg was injected by bolus through one of the arm veins followed by a 10-mL saline flush.					
**Langhorst 2007**	CD&UC (29)	>18	Prospective	Endoscopy	to compare a faecal-tagging based MR colonography to colonoscopy	-	A solution containing 5% Gastrografin (Schering, Germany), 1% barium (Polibar, EZ-EM, Westbury, NY), and 0.2% locust bean gum (Roeper, Germany) was ingested in portions of 250 mL	a 1.5 T MR system (Magnetom Sonata, Siemens Medical Solutions, Germany).		63%		TrueFISP images and T1w gradient echo images, which were acquired pre- and post-intravenous contrast (0.2 mmol/kg body weight of Gd-DOTA; Dotarem, Guerbet, France)

Study ID	IBD Type (n)	Age	Study Design	Reference Standard	Aims and Outcomes	Disease Scoring System	Oral Contrast	Modality/Manufacturers	Accuracy (%)	Sensitivity (%)	Specificity (%)	Modality Protocol
							with each regular meal starting 48 hours prior to the MR examination.					
**Koh 2001**	CD (30)	18 to 58 years	Prospective	Endoscopy	to evaluate the sensitivity and specificity of MR imaging in assessing the activity of Crohn's disease.	Crohn's disease activity index (CDAI)	All patients drank 600 mL of water 30 min before the examination. IV contrast injection was given	1.0 T MRI (Magnetom Impact; Siemens, Erlangen, Germany)	-	91.0	71.0	coronal plane using a T2-weighted turbo spin-echo sequence, T1-weighted fast low-angle shot imaging in the coronal, axial, and sagittal planes, a T1-weighted fast low-angle shot sequence for after contrast injection in sagittal and coronal planes.
**Mostafa 2021**	UC (30)	18 to 59 years	Prospective	Endoscopy and histopathology	to detect this change in colonic wall thickness of the sigmoid colon	Truelove and Witt's' classification	-	US	80.0	83.0	77.0	-
**Lenze 2012**	CD (30)	22 to 63 years	prospective	Endoscopy and histology	to assess the best non-invasive imaging	Crohn's Disease Activity	2000 mL of a water mixed oral contrast	Abdominal US (using 3.5–5 MHz convex transducer and 7 MHz linear	-	-	-	The MR protocol that was used involved both axial and coronal T2-

Study ID	IBD Type (n)	Age	Study Design	Reference Standard	Aims and Outcomes	Disease Scoring System	Oral Contrast	Modality/Manufacturers	Accuracy (%)	Sensitivity (%)	Specificity (%)	Modality Protocol
					method for the detection and differentiation of inflammatory and fibromatous stenoses in CD in comparison to endoscopic and histologic evaluation.	Index (CDAI)	with methylcellulose solution	transducer), MR-Enteroclysis and one fluorine 18-labelled fluoro-2-deoxy-D-glucose (^{18}FDG) / positron emission tomography (PET)/CT				weighted sequence and fat saturation T1-weighted sequence after contrast injection was administered.
**Fiorino 2013**	CD (32)	20 to 72 years	Prospective	Ileocolonoscopy	Detecting CD lesions	CD endoscopic index of severity (CDEIS)	1500 mL of polyethylene glycol electrolyte solution (PEG, Colirei, ABC Farmaceutici Spa, Torino, Italy)	1.5T MRI Symphony scanner (Siemens, Erlangen, Germany)	86.0	81.0	75.0	Both the axial and coronal planes of fat-saturated T1-weighted images were performed at the beginning of the 3T MRI session.
								3T MRI (Verio scanner (Siemens, Erlangen, Germany)	93.0	86.0	100	
					Bowel wall thickening			1.5T MRI	80.0	-	-	-
								3T MRI	92.0	-	-	
					Detecting strictures			1.5T MRI	80.0	-	-	-
								3T MRI	90.0	-	-	
					Detecting ulcerations			1.5T MRI	42.0	-	-	-
								3T MRI	76.0	-	-	

Study ID	IBD Type (n)	Age	Study Design	Reference Standard	Aims and Outcomes	Disease Scoring System	Oral Contrast	Modality/Manufacturers	Accuracy (%)	Sensitivity (%)	Specificity (%)	Modality Protocol
**Maccioni 2014**	CD (50)	6 to 18 years	Prospective	Ileocolonoscopy	Detecting CD lesions	-	200-700 mL of (Lumirem, Guerbet, Paris, France)	MRI 1.5T (Magnetom Avanto scanner (Siemens Healthcare)	-	86.8	94.5	using T2-weighted single-shot turbo SE, fast SE with both fat and not fat saturation, and T1W 3D gradient-echo sequences, with gadolinium as an intravenous contrast agent.
**Gaitini 2011**	CD (52)	9 to 80 years	Prospective	Multidetector CT ((Brilliance 64-channel scanner or Brilliance 16 slice scanner; Philips Healthcare)	Assessing Disease Extension	-	between 1500 to 2000 mL (Telebrix; Geurbet, Roissy, France)	US (HDI 5000 or iU22 equipment (Philips Healthcare, Bothell, WA)	83.0	67.0	85.0	All the sonographic examinations were performed with a linear array 4 to 8 or 5 to 12 MHz probe.
**Maccioni 2006**	CD (59)	18 to 76 years	Prospective	Ileocolonoscopy	Detecting CD lesions	-	600-900 mL of Lumirem (Laboratoires Guerbet, Paris, France)	MRI 1.5 (Magnetom Vision Plus; Siemens)	94.0<br>93.5	76.3<br>76.3	98.0<br>97.0	involved T1-weighted fast low-angle shot (FLASH) sequences and T2-weighted half-Fourier rapid acquisition with relaxation enhancement RARE sequences with and without fat suppression and

Study ID	IBD Type (n)	Age	Study Design	Reference Standard	Aims and Outcomes	Disease Scoring System	Oral Contrast	Modality/Manufacturers	Accuracy (%)	Sensitivity (%)	Specificity (%)	Modality Protocol
												before and after contrast.
**Allocca 2018**	CD (60)	>18	Prospective	Endoscopy (SES-CD)	to compare for the first time the accuracy of bowel US alone versus MRE + CS together [as a reference standard] in assessing disease activity and complications in CD	Simple Endoscopic Score for CD (SES-CD)	Neither preparation nor contrast was used	using an Aloka Arietta V60 with convex [5–1 MHz] and microconvex probes [4–8 MHz].	96	92	100	-
**Pascu 2004**	Total (61)<br>CD (37)<br>UC (24)	19-84	Prospective	Ileocolonoscopy	Assessing Disease Extension (Overall, CD, and UC, respectively)	The modified Baron score	-	US (9 and 11 MHz linear array and a 7 MHz matrix transducer on a GE Logiq 700 scanner (General Electrics) 1.5T MRI (S15 ACS, Philips Medical System)	88.3<br>83.3<br>96.3<br>70.0<br>63.5<br>79.8	79.7<br>67.5<br>97.0<br>45.7<br>29.5<br>63.3	96.3<br>96.5<br>97.5<br>89.7<br>88.3<br>92.3	involved Gadolinium-enhanced turbo spin-echo sequence
**Vigano 2019**	CD (65)	15 to 79	Prospective	Pathological data	Detecting CD lesions	-	-	IOUS (ProSound Alpha 5sv or ProSound	100	100	-	-

Study ID	IBD Type (n)	Age	Study Design	Reference Standard	Aims and Outcomes	Disease Scoring System	Oral Contrast	Modality/Manufacturers	Accuracy (%)	Sensitivity (%)	Specificity (%)	Modality Protocol
								Alpha 7 ultrasound system (Hitachi Aloka Medical, Tokyo, Japan, with a linear multi-frequency (5-10 MHz) probe) US<br>MRI	79.4<br>81.3	84.2<br>86.1	-<br>-	
					Detecting stenoses			IOUS<br>US<br>MRI	100<br>83.8<br>83.8	100<br>86.8<br>86.8	-<br>-<br>-	-
					Detecting fistulas			IOUS<br>US<br>MRI	98.5<br>92.3<br>92.3	93.8<br>75.0<br>81.3	-<br>-<br>-	-
**Yuksel 2019**	CD (71)	18 to 63	Prospective	Ileocolonoscopy	Detecting CD disease (Bowel wall thickening)	Simple Endoscopic Score for CD (SES-CD)	-	US (GE LOGIC S6 device (GE Healthcare, Chicago, Illinois, USA) using a convex probe (3.5 – 5.5 MHz) at the beginning and followed by a linear probe (7–12MHz)<br>1.5T MRI (Siemens, Erlangen, Germany)	77.5<br>74.4	49.3<br>54.9	91.5<br>84.2	-

Study ID	IBD Type (n)	Age	Study Design	Reference Standard	Aims and Outcomes	Disease Scoring System	Oral Contrast	Modality/Manufacturers	Accuracy (%)	Sensitivity (%)	Specificity (%)	Modality Protocol
					Detecting CD disease (Stenosis)			US<br>MRI	71.4<br>73.0	16.9<br>35.2	98.6<br>91.9	
**Haber 2002**	Total 78<br>CD (26)<br>UC (21)<br>Indetermined and others (31)	1 month to 17.8 years	Prospective	Endoscopy and histopathology	to determine whether ultrasonography can be useful in assessing the extent of the disease in a series of children and young adults with CD, ulcerative colitis (UC), and various other intestinal disorders and to compare ultrasonographic findings with clinical, endoscopic, and histologic data	CD: Pediatric Crohn's Disease Activity Index (PCDAI)<br>UC: according to the criteria used by Edwards and Truelove	-	US	-	77.0 s depicted on endoscopy<br>75.0 s depicted on histopathology	83.0 s depicted on endoscopy<br>82.0 s depicted on histopathology	-
**Oussalah 2010**		age 24 to	Prospective	Colonoscopy	Detection of Endoscopic	The modified	-	1.5T MRI	-	89.5	87.0	-

Study ID	IBD Type (n)	Age	Study Design	Reference Standard	Aims and Outcomes	Disease Scoring System	Oral Contrast	Modality/Manufacturers	Accuracy (%)	Sensitivity (%)	Specificity (%)	Modality Protocol
	Total (96)<br>CD (61)<br>UC (35)	45 years			Inflammations UC group	Baron score/ Simple Clinical Colitis Activity Index (SCCAI)/ Simple Endoscopic Score for CD (SES-CD)		(GE Signa HDx scanner (General Electric Healthcare, Waukesha, Wisconsin, USA)				
					Detection of Endoscopic Inflammations CD group				-	58.0	84.5	-
**Jesuratnam-Nielse 2015**	Total (100)<br>44 (CD),<br>25 (UC)<br>24 with IBD unclassified<br>7 had other diagnosis	19 to 90	Prospective	MRI with oral bowel prep	Detecting CD lesions	-	1350 mL of VoLumen (Bracco Diagnostics, Milan, Italy	MRI (Achieva scanner (Philips Healthcare, Best, the Netherlands)	92.0	71.0	93.0	-
					Detecting UC lesions			MRI	86.0	40.0	90.0	-
**Jesus Martinez 2019**	CD (108)		Prospective	Colonoscopy	Assessing Disease Extension	-	-	US	90.7	-	-	-
**Limberg 1989**	CD and UC (142)	>18	Prospective	colonoscopy	To investigate whether the retrograde instillation of fluid into the	Crohn's disease activity index	-	US		91.0 (CD)<br>89.0 (UC)	100.0 (CD)<br>97.0 (UC)	

Study ID	IBD Type (n)	Age	Study Design	Reference Standard	Aims and Outcomes	Disease Scoring System	Oral Contrast	Modality/Manufacturers	Accuracy (%)	Sensitivity (%)	Specificity (%)	Modality Protocol
					colon would also improve the diagnostic value of ultrasonography in evaluating and differentiating inflammatory large bowel disease	(CDAI) for CD						
**Taylor 2019**	CD (284) New diagnosis (133) Suspected relapse (151)	>16	Prospective	Ileocolonoscopy	Colonic CD Presence in newly diagnosed cohort	-	-	US<br>MRI	-<br>-	52.0<br>39.0	86.0<br>94.0	a minimal protocol data set compromising both fat-saturated and non-fat-saturated T2-weighted, gradient echo, DWI before contrast and T1-weighted sequence after injecting the gadolinium contrast agent.
					Colonic CD Presence in relapsed cohort			US<br>MRI	-<br>-	41.0<br>47.0	93.0<br>95.0	
					Colonic CD activity in newly diagnosed cohort			US<br>MRI	-<br>-	46.0<br>40.0	94.0<br>93.0	
					Colonic CD activity in relapsed cohort			US<br>MRI	-<br>-	37.0<br>55.0	95.0<br>95.0	

Table S3. QUADAS-2 summary of risk and applicability (tabular format).

Study First Author, Year	Risk of Bias				Applicability Concerns		
	Patient Selection	Index Test	Reference Standard	Flow and Timing	Patient Selection	Index Test	Reference Standard
Dagia 2008 [38]	U	U	U	H	U	U	U
Laurent 2017 [16]	L	H	L	L	L	H	L
Dillman 2011 [28]	L	L	L	L	L	L	L
Campari 2017 [34]	H	H	H	H	H	L	H
Kamel 2020 [21]	L	L	H	H	L	L	H
Cansu 2020 [18]	L	L	H	L	L	L	H
Barber 2017 [53]	L	L	H	L	L	L	H
Liu 2021 [27]	L	L	L	L	L	L	L
Johnson 2009 [54]	L	L	H	H	L	L	H
Kim 2019 [47]	L	L	H	H	L	L	H
Thierry 2018 [55]	L	L	L	L	L	L	L
Wilkens 2016 [46]	L	U	L	L	L	U	L
Schreyer 2011 [39]	L	U	U	L	L	L	U
Paparo 2012 [56]	L	L	L	L	L	L	L
Kawai 2019 [40]	H	L	H	L	H	L	H
Podgorska 2021 [23]	L	L	L	L	L	L	L
Boraschi 2016 [35]	L	H	H	H	L	L	H
Yu 2015 [15]	L	L	L	L	H	L	L
Ponorac 2021 [57]	L	L	L	L	L	L	L
Langhorst 2007 [41]	L	L	H	H	L	L	H
Koh 2001 [20]	L	L	H	H	L	L	H
Mostafa 2021 [24]	L	L	H	H	L	L	H
Lenze 2012 [50]	L	L	L	L	L	L	L
Fiorino 2013 [17]	L	L	L	L	L	L	L
Maccioni 2014 [29]	L	L	H	L	L	L	H
Gaitini 2011 [36]	L	H	L	L	L	H	L
Maccioni 2006 [30]	L	L	L	L	L	L	L
Allocca 2018 [26]	L	L	L	L	L	L	L
Pascu 2004 [14]	L	L	H	L	H	L	H
Vigano 2019 [32]	L	H	H	L	L	H	H
Yuksel 2019 [25]	L	L	L	L	L	L	L
Haber 2002 [22]	H	L	H	H	H	L	H
Oussalah 2010 [13]	L	L	L	L	L	L	L
JESURATNAM-NIELSEN 2015 [37]	L	H	L	L	L	H	L
Jesus Martinez 2019 [33]	L	L	L	L	L	L	L
Limberg 1989 [19]	L	L	L	U	L	L	H
Taylor 2019 [31]	L	L	L	L	L	L	L

L = low risk of bias; **U** = unclear risk of bias; **H** = high risk of bias.
